# Supplementary material for: Assessment of Water, Sanitation, and Hygiene Conditions in Public Elementary Schools in Quetzaltenango, Guatemala, in the Context of the COVID-19 Pandemic
Source: Int J Environ Res Public Health. 2023 Oct 13;20(20):6914. doi: 10.3390/ijerph20206914 (PMC10606716; doi:10.3390/ijerph20206914)
Supplement: Supplementary file 1 [file ijerph-20-06914-s001.zip › ijerph-2575781-supplementary.pdf]

SUPPLEMENTARY MATERIAL

Table S1. JMP WASH service levels of the six participating schools.

School	Drinking water	Sanitation	Hygiene
**1**	Basic service	Limited service	Basic service
**2**	Basic service	Limited service	Basic service
**3**	Basic service	Limited service	Limited service
**4**	Basic service	Limited service	Basic service
**5**	Basic service	Limited service	Basic service
**6**	Basic service	Limited service	Basic service
